# Non-invasive assessment of embryo development and ploidy: morphokinetics predict blastulation, and exploratory metabolomics identifies candidate metabolic signatures for euploidy

**DOI:** 10.3389/fcell.2026.1914948

**Published:** 2026-09-18

**Authors:** Xuehua Chen, Ruoyun Lin, Jinhui Shu, Huanhua Chen, Kejian Sun, Xin Zhao, Zhan Li, Zhulian Wu, Hong Zhou, Caizhu Wang

**Affiliations:** Center of Reproductive Medicine, Maternity and Child Healthcare of Guangxi Zhuang Autonomous Region, Nanning, Guangxi, China

**Keywords:** embryo selection, euploid, kinetic parameters, metabolomics, noninvasive assessments

## Abstract

**Purpose:**

To identify more reliable tools for embryo selection, this study aimed to investigate whether embryonic kinetic parameters and metabolomic profiling of spent culture medium on D3 can predict subsequent blastocyst development and chromosomal euploidy.

**Methods:**

This prospective diagnostic study analyzed 1,260 embryos from 111 PGT-M or PGT-A patients (April 2024–July 2025) cultured in time-lapse incubators. For the morphokinetic analysis, generalized linear mixed models with patient ID as a random effect were used to identify kinetic parameters associated with blastocyst formation, and the final model was internally validated using a 70/30 hold-out split. Additionally, metabolomic profiling of D3 spent culture medium from 33 patients was performed using LC-MS, with features prioritized by VIP >1.0 and FDR controlled via the Benjamini–Hochberg procedure for multiple comparisons.

**Results:**

Our results delineated specific abnormal cleavage patterns (Direct cleavage, Uneven blastomere, Blastomere fragmentation) and most kinetic parameters (all except t3 and t5) were significantly associated with usable blastocyst formation potential (*P < 0.05)* but showed no association with ploidy status (*P > 0.05*). Optimal developmental range was identified for each parameter, and embryos developing within these windows demonstrated significantly higher usable blastocyst formation rates (*P* < 0.001). Our model demonstrated good performance in the training set (AUC = 0.826) and maintained acceptable discrimination in the validation set (AUC = 0.724), comparable to the KID score (AUC = 0.738; DeLong test *P* = 0.682). In contrast, metabolomic screening identified 86 VIP-ranked features; stringent FDR correction rendered none significant (all adjusted *P-value > 0.05*). Based on endogenous retention among the VIP-ranked features, 11 metabolites were retained as exploratory candidates; six of which—sphingosine, phytosphingosine, N,N-dimethylsphingosine, histidylproline, L-valine, and indoleacetaldehyde—displayed AUC >0.7 trends (pooled n = 17 euploid, 10 aneuploid) requiring independent validation.

**Conclusion:**

Our results demonstrate that early kinetic parameters robustly predict blastocyst formation, whereas D3 SCM metabolomics provides only tentative, effect-size-dependent clues for ploidy status that require cautious interpretation. These complementary findings lay the groundwork for a future integrated noninvasive assessment strategy on D3.

## Introduction

The advent of *in vitro* fertilization (IVF) in 1978 marked a transformative milestone in the treatment of infertility (Steptoe et al., 1978). While assisted reproductive technology (ART) has since achieved considerable advances, a significant consequence has been an increased incidence of multiple pregnancies, elevating associated maternal and neonatal risks (Practice Committee of the Society for Reproductive Endocrinology and Infertility, 2022; Wang et al., 2021). In response, elective single embryo transfer (eSET) has been widely advocated to mitigate these complications (Ubaldi et al., 2015; Mejia et al., 2020). A critical determinant of eSET success is the selection of a single embryo with the highest developmental potential. However, extended culture to the blastocyst stage, often preferred for selection, carries inherent limitations, including the risk of cycle cancellation if blastulation fails and potential epigenetic alterations (White et al., 2015; Sirard, 2017). Consequently, selecting the optimal embryo for transfer at the cleavage stage presents a compelling alternative, provided that a highly accurate method for predicting embryonic developmental potential is available.

Accurate embryo selection depends not only on morphological competence but, fundamentally, on chromosomal normality. Aneuploidy remains a leading cause of implantation failure, miscarriage, and congenital abnormalities (Li et al., 2022). Although preimplantation genetic testing for aneuploidies (PGT-A) represents the current gold standard for ploidy assessment, its clinical application is constrained by invasiveness, substantial cost, and diagnostic uncertainties attributable to embryonic mosaicism (Zheng et al., 2021; Zhang et al., 2020). These limitations underscore the pressing need for a robust, noninvasive methodology capable of identifying euploid embryos with high developmental potential early in development, ideally by day 3 (D3), thereby circumventing the need for extended culture and trophectoderm biopsy. Supporting the biological plausibility of early assessment, accumulating evidence indicates that the developmental competence of human embryos is largely determined by molecular and cellular characteristics established prior to embryonic genome activation (Wong et al., 2010).

Conventional morphological grading offers limited predictive value due to its subjective and static nature (Jiang et al., 2022). Time-lapse imaging (TLI) has emerged as a superior alternative, enabling continuous, quantitative analysis of dynamic kinetic parameters (ESHRE Working g roup on Time-lapse technology et al., 2020). Several studies suggest that D3 morphokinetics may predict developmental competencies such as blastocyst formation and implantation potential (Milewski et al., 2015; Carrasco et al., 2017; Kalyani and Deshpande, 2024). However, there is marked heterogeneity in both the parameters selected and the performance of prediction models across studies, limiting widespread clinical adoption is not yet widespread (Kalyani and Deshpande, 2024; Herrero et al., 2013; Pennetta et al., 2021; Liu et al., 2016). More critically, evidence regarding the correlation between kinetic parameters and embryonic ploidy is both limited and conflicting, with significant overlap in the development timelines of euploid and aneuploid embryos (Pennetta et al., 2021; Bamford et al., 2023; Rajendran et al., 2024; Giménez et al., 2023). Moreover, few studies have comprehensively assessed whether early kinetic parameters can further discriminate embryonic euploidy beyond predicting blastocyst formation potential. Thus, whether kinetics can reliably serve as a noninvasive proxy for PGT-A remains an open question.

Metabolomic profiling of spent culture medium (SCM) presents another promising, noninvasive avenue for assessing embryonic viability. The metabolome reflects the functional output of genomic and proteomic activity, potentially offering a direct readout of embryonic physiology. Preliminary evidence indicates that euploid and aneuploid embryos exhibit distinct metabolic signatures in their culture environment (Eldarov et al., 2022), suggesting that exometabolomics analysis could offer functional insights into developmental competence and enhance the efficacy of single cleavage embryo transfer. Nevertheless, a direct, head-to-head comparison of the predictive performance between metabolomic markers and well-defined kinetic parameters remains lacking, impeding the development of an integrated noninvasive assessment strategy.

Therefore, this study was designed to conduct a rigorous comparative evaluation of these two noninvasive approaches. We aimed to systematically evaluate the ability of early kinetic parameters to predict both blastocyst formation and embryonic euploidy. Concurrently, untargeted metabolomic analysis of D3 SCM was employed to identify ploidy-specific metabolic markers and associated biological pathways. Based on the hypothesis that kinetic and metabolomic data provide complementary information, our work lays a solid foundation for developing an integrated, noninvasive strategy to assess the developmental potential of day 3 embryos.

## Materials and methods

### Study design and participants

This prospective cohort study was conducted at a single reproductive medical center between 1 April 2024, and 31 July 2025. The study included infertile patients undergoing preimplantation genetic testing for monogenic (PGT-M) or PGT-A. To minimize confounding factors in embryo development assessment, strict inclusion and exclusion criteria were applied. Eligible patients were women age ≤38 years who had ≥8 oocytes retrieved and produced at least five two-pronuclei (2  P N) zygotes in a single cycle. Exclusion criteria encompassed cycles with failure to form any blastocysts available for biopsy, patients carrying chromosomal structural rearrangements (e.g., balanced translocations, Robertsonian translocations, or inversions), and cycles where sperm was obtained through surgical procedures (including testicular sperm aspiration or microdissection testicular sperm extraction).

### Controlled ovarian stimulation

Ovarian stimulation was performed using either a long protocol or an antagonist protocol, with the selection individualized based on patient characteristics. Among the included cycles, the long protocol was employed in 80 cases, primarily for patients with endometriosis/adenomyosis or heterogeneous follicular development to improve follicular synchronization. The antagonist protocol was used in 31 cases, favored for patients at high risk of ovarian hyperstimulation syndrome, as well as for cost-sensitive individuals or those with uniform follicular cohorts at baseline.

Long Protocol: A gonadotropin-releasing hormone agonist (GnRH-a; triptorelin 0.1 mg/d or 0.05 mg/d, Decapeptyl, Ferring, Germany) was administered subcutaneously starting in the mid-luteal phase. After 14–21 days, recombinant follicle-stimulating hormone (r-FSH; Gonal-F, Merck Serono, Switzerland) was initiated at a daily dose of 150–300 IU/d once pituitary downregulation was confirmed, defined as serum FSH and luteinizing hormone (LH) <5 IU/L and estradiol (E2) <50 pg/mL. The GnRH-a was continued until the day of trigger. Final oocyte maturation was triggered with human chorionic gonadotropin (HCG; 5,000–10,000 IU; Serono, Switzerland or Livzon, China) when at least three follicles reached a mean diameter of ≧18 mm.

Antagonist Protocol: Ovarian stimulation with r-FSH was initiated on menstrual cycle days 2–3. A GnRH antagonist (Cetrotide, MSD China) was introduced when the leading follicles reached 11–13 mm, supplemented by serum criteria with LH ≥ 10 IU/L or E2 ≥ 1,570 pmol/L. Triggering with HCG (6000–8000 IU) was performed when ≥3 follicles reached ≥17 mm or ≥2 follicles reached ≥18 mm.

### Intracytoplasmic Sperm Injection (ICSI) and embryo culture

Oocytes were retrieved transvaginally 34–36 h after HCG administration. Retrieved oocyte-cumulus complexes were denuded using hyaluronidase, and ICSI was performed 3–4 h after retrieval, exclusively on metaphase II (MII) oocytes. Semen samples were processed using density gradient centrifugation (80% and 40%, Sperm Gradient, Quinn Advantage Medium, ART-2040, Sage) or the swim-up technique before injection.

Fertilization was assessed 16–18 h post-ICSI. Only 2PN zygotes were cultured in a time-lapse incubator (EmbryoScope®, Vitrolife, Sweden) at 37 °C under tri-gas conditions (89% N_2_, 5% O_2_, 6% CO_2_). Cleavage medium (Quinn’s Advantage Medium, ART-1026, Sage) supplemented with 10% serum protein substitute (SPS) was used from D1 to D3. On D3, embryos were transferred to blastocyst medium (Quinn’s Advantage Medium, ART-1029, Sage) supplemented with 10% SPS and cultured continuously until D5, 6, or seven within the same time-lapse system.

### Time-lapse monitoring and kinetic parameter analysis

Embryo development was continuously monitored using the EmbryoScope® time-lapse system. Key early kinetic parameters were recorded up to D3, including the time of pronuclear fading (tPNf) and the time points of cleavage to 2, 3, 4, 5, and 8 discrete blastomeres (t2, t3, t4, t5, t8). From these measurements, the following durations were calculated: cc1 (t2-tPNf), cc2 (t3-t2), s2 (t4-t3), s3 (t8-t5).

Concurrently, abnormal cleavage patterns were documented throughout early development and defined as follows.

- Direct cleavage (DC): immediate division of one blastomere into three or more daughter cells.
- Reverse cleavage (RC): fusion of two or more blastomeres into a single cell.
- Uneven blastomere (UB): division resulting in two daughter blastomeres of significantly different size.
- Multinucleation (Mn): presence of two or more cell nuclei with a single blastomere.
- Blastomere fragmentation (BF): immediate disintegration of a blastomere into fragments during the cleavage process.

All observations were restricted to the cleavage stages (1–8 cell stage). And all morphokinetic annotations and assessments of abnormal cleavage patterns were performed on D3, prospectively prior to blastocyst formation and PGT biopsy, by a single experienced embryologist who was blinded to blastocyst grading and ploidy outcomes. A schematic representation of the early embryonic development timeline and kinetic parameters is provided in Figure 1.

**FIGURE 1:**
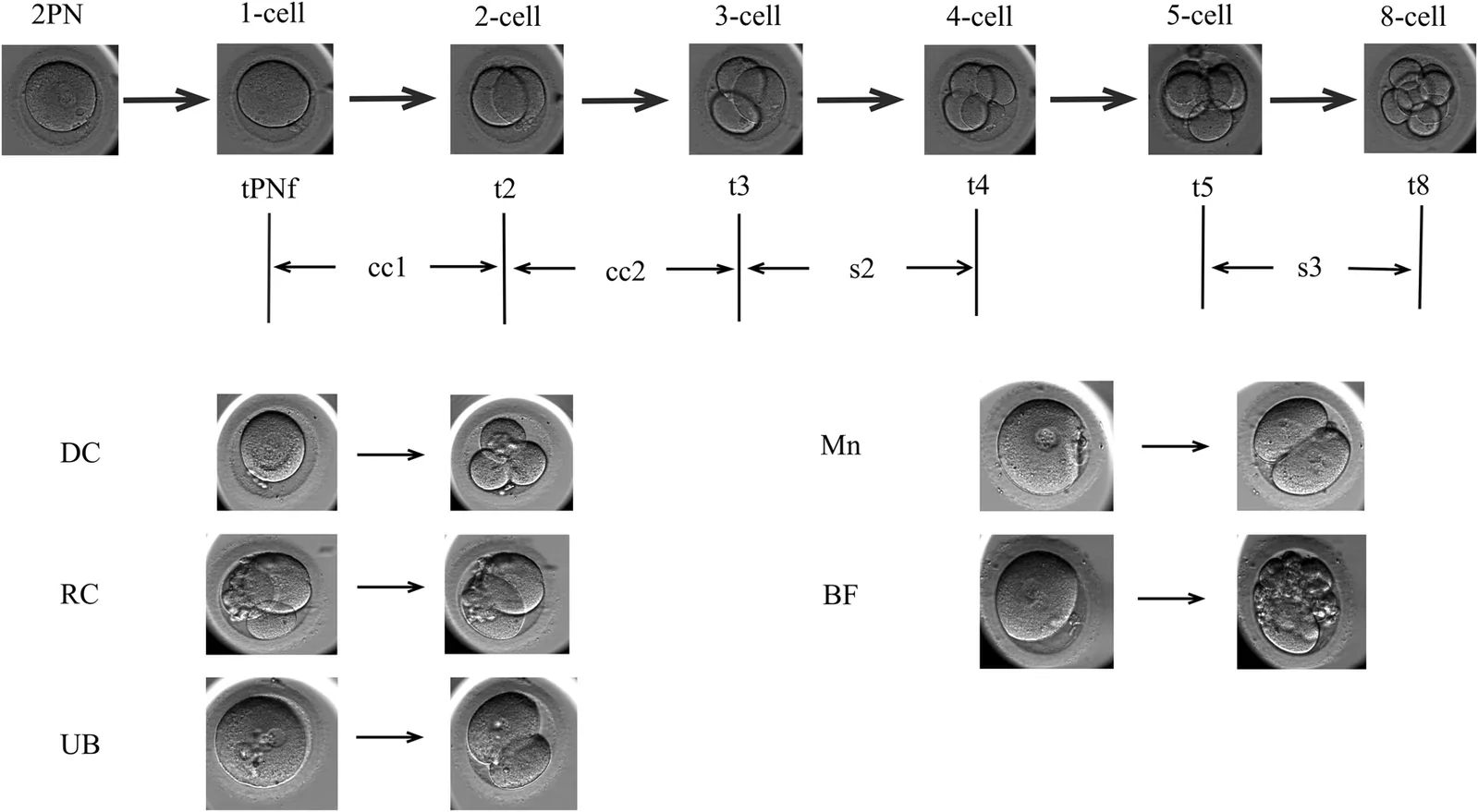
Early embryonic development timeline, kinetic parameters and abnormal cleavage patterns. Note: The time of pronuclear fading (tPNf), time of cleavage to 2, 3, 4, 5, and 8 discrete blastomeres (t2, t3, t4, t5, t8) and cc1 (t2-tPNf), cc2 (t3-t2), s2 (t4-t3), s3 (t8-t5). DC, direct cleavage; RC, reverse cleavage; UB, uneven blastomere; Mn, multinucleation; BF, blastomere fragmentation. The figure depicts abnormal cleavage patterns from only one stage of the division process; patterns from other stages are not presented.

**FIGURE 2:**
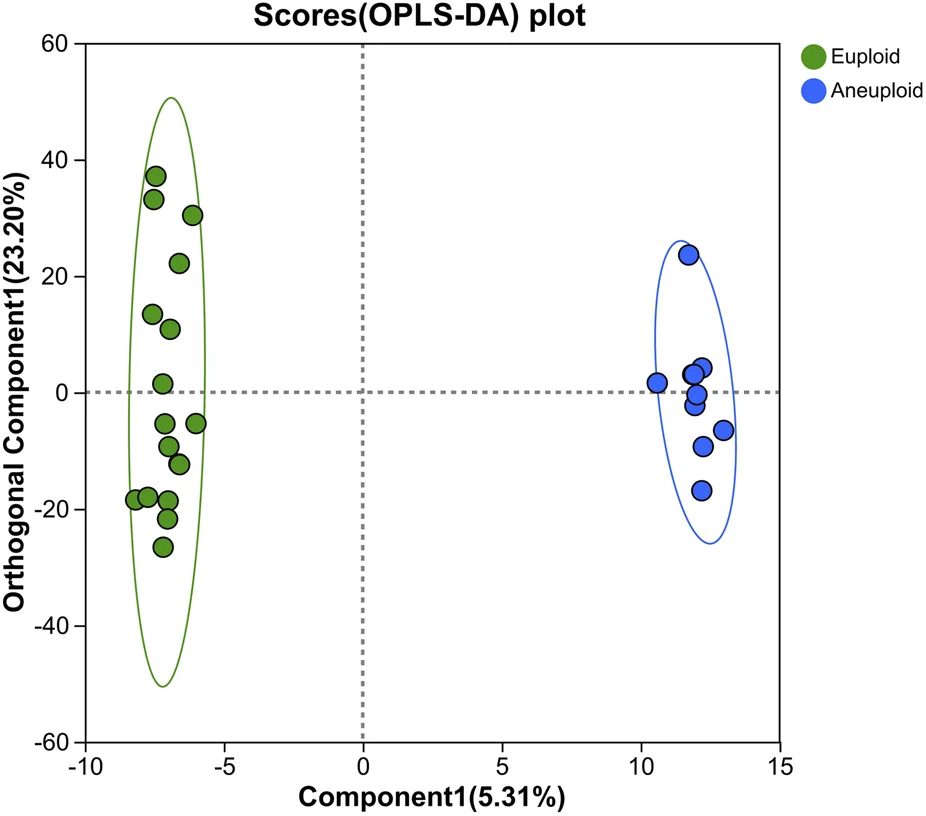
OPLS-DA score plot derived from the metabolomic profiles of D3 SCM. Note: Component 1 (5.31%) represents the predictive variance that separates embryos by ploidy status, while orthogonal component 1 (23.20%) reflects systematic variation unrelated to grouping. Each point represents an individual SCM sample (n = 27 samples).

### Blastocyst grading and biopsy

Blastocysts were graded according to the Gardner criteria (Gardner et al., 2000), which evaluate the degree of expansion (1–6) and the quality of both the inner cell mass (ICM) and trophectoderm (TE) (grades A-C). Based on this grading, blastocysts were categorized as follows: usable blastocysts exhibited expansion ≥3 with ICM/TE grades of at least BC/CB; high-quality blastocysts demonstrated expansion≥3 with ICM/TE grades of AA, AB/BA, or BB. Those with expansion 1–2 or a grade C for either ICM or TE were classified as low-quality. The usable and high-quality blastocyst rates were calculated as the respective number divided by the total number of 2PN zygotes.

All usable blastocysts (≥3BC/CB) were selected for TE biopsy. The procedure was performed in a biopsy dish containing Quinn’s Advantage Medium (ART-1023, Sage, United States of America) supplemented with 10% SPS. The blastocyst was immobilized, and a laser pulse was applied to create an opening in the zona pellucida away from the ICM. Approximately 5–8 TE cells were gently aspirated through the aperture, separated using a laser, and transferred into a sterile tube for storage at −20 °C for genetic analysis. Following biopsy, all blastocysts were vitrified and stored in liquid nitrogen.

### Genetic analysis

All trophectoderm biopsy samples were subjected to comprehensive chromosomal analysis by next-generation sequencing (NGS). Whole-genome amplification (WGA) was performed using the Universal Sample Processing kit (Yikon Genomics, Shanghai, China), followed by library construction with the manufacturer’s Universal DNA Fragmentation Kit. Sequencing was conducted on an Illumina HiSeq 2,500 platform (Illumina, San Diego, CA, United States of America) under 2 × 100 bp paired-end mode. Chromosomal copy number variations (CNVs) were analyzed using ChromGO software (Yikon Genomics). Samples with intermediate chromosomal copy numbers between 30% and 70% were classified as mosaic, whereas those below 30% or above 70% were categorized as euploid or aneuploid, respectively.

For cases undergoing PGT-M**,** a family-specific haplotype-based NGS assay was employed, which concurrently detected the targeted monogenic/single-gene disorder and screened for chromosomal aneuploidy (PGT-A). For PGT-A only cases, the standard NGS-based comprehensive chromosome screening protocol, as described above, was applied. Based on the genetic results, embryos were categorized as euploid, aneuploid, mosaic or segmental aneuploid.

### Data analysis

Continuous variables were tested for normality using the Shapiro-Wilk test. Data conforming to a normal distribution were analyzed using Student’s t-test, while the Mann-Whitney U-test was applied for non-normally distributed data. Categorical variables were compared using the Chi-square test. To account for intra-patient correlation, generalized linear mixed models (GLMM) with patient ID as a random intercept were used for all kinetic analyses, with a binomial distribution and logit link function for the binary outcome (usable blastocyst formation). Variables with *P* < 0.1 in the univariate analysis were entered into the multivariate GLMM. Multicollinearity was assessed using variance inflation factors (VIF). Internal validation was performed using a 70/30 hold-out split, and model performance was evaluated by AUC in the validation set. The predictive performance of our model was compared with the KID score using DeLong’s test for paired AUCs. A two-sided *P* < 0.05 was considered statistically significant. All analyses were performed using SPSS software (version 27.0; IBM Corp.).

## Metabolomic profiling

### Sample collection and preparation

A total of 376 D3 SCM samples were prospectively collected from 33 enrolled PGT-M/PGT-A cycles between March 5 and 8 June 2025. Samples were immediately frozen in liquid nitrogen and stored at −80 °C until analysis. According to the PGT results, there were a total of 68 euploid and 40 aneuploid embryos, and embryos with mosaic or segmental abnormalities were excluded to enable a dichotomous comparison. To increase metabolite detection sensitivity, four individual samples were pooled into one tube prior to analysis. Pooling was performed across different patients based on their ploidy outcomes (not within-patient), ensuring independence of the pooled samples. This pooling strategy yielded euploid group pooled samples (n = 17) or aneuploid group (n = 10).

Metabolites were extracted using acetonitrile: methanol (1:1, v/v) containing the internal standard L-2-chlorophenylalanine. The extraction procedure included vortexing, sonication (5 °C, 40 kHz), incubation at −20 °C, and centrifugation (13,000 g, 4 °C, 15 min). The supernatant was dried under nitrogen and re-dissolved in acetonitrile: water (1:1, v/v) for subsequent analysis.

### Liquid Chromatography-Tandem Mass Spectrometry (LC-MS) analysis

Metabolomic profiling was performed using a Thermo UHPLC-Q Exactive HF-X system equipped with an ACQUITY HSS T3 column. The mobile phases consisted of solvent A (0.1% formic acid in water: acetonitrile, 95:5) and solvent B (0.1% formic acid in acetonitrile: isopropanol: water, 47.5:47.5:5). Chromatographic separation was conducted at a flow rate of 0.40 mL/min and a column temperature of 40 °C.

Mass spectrometry detection was performed in both positive and negative electrospray ionization modes with the following parameters: source temperature, 425 °C; sheath gas, 50 arb; auxiliary gas, 13 arb; ion spray voltage, ±3500 V; collision energy, 20–60 V. Full MS scans were acquired at a resolution of 60,000 and MS/MS scans at 7,500, covering a mass range of 70–1,050 m/z.

### Metabolomic data processing and statistical analysis

Raw LC-MS data were processed using Progenesis QI software (Waters Corporation, Milford, United States of America) to generate a feature matrix. After the removal of internal standards, known artifacts, and redundant features, metabolite annotation was performed by searching against the HMDB (http://www.hmdb.ca/), Metlin (https://metlin.scripps.edu/), and Majorbio Database (MJDB) of Majorbio Biotechnology Co. Ltd. (Shanghai, China). Metabolite identification was assigned with Level 2 confidence (putative annotation) according to the Metabolomics Standards Initiative (MSI) guidelines, based on accurate mass (≤5 ppm) and retention time matching against the above databases. The data was uploaded to the Majorbio cloud platform (https://cloud.majorbio.com) for data analysis. Firstly, the data of the metabolic features matrix was pre-processed, as follows: at least 80% detected in any set of samples were retained. After filtering, the minimum value in the data matrix was selected to fill the missing value, and each metabolic to reduce the errors caused by sample preparation and instrument instability, the response intensities of the sample mass spectrometry peaks were normalized using the sum normalization method to obtain the normalized data matrix. Meanwhile, the variables of QC samples with relative standard deviation (RSD) > 30% were excluded, and log10 logarithmicized to obtain the final data matrix for subsequent analysis. Perform variance analysis on the matrix file after data preprocessing.

Multivariate statistical analyses, including principal component analysis (PCA), partial least squares-discriminant analysis (PLS-DA) and orthogonal partial squares discriminant analysis (OPLS-DA) were performed using the “ropls” package in R (v1.6.2). Model robustness was evaluated via 7-fold cross-validation. Features with VIP >1.0 and P < 0.05 were considered statistically significant. The FDR-adjusted *P* (Benjamini–Hochberg procedure) were also reported to account for multiple comparisons. Pathway enrichment analysis was conducted using the KEGG database (http://www.genome.jp/kegg/), with significance determined by a right-tailed Fisher’s exact test (*P* < 0.05).

## Results

### Patient demographics and embryo development outcomes

A total of 111 PGT cycles (82 PGT-M and 29 PGT-A) were included. The study population had a mean female age of 32.47 ± 3.83 years, a mean body mass index (BMI) of 21.97 ± 2.65 kg/m2, and an average infertility duration of 2.05 ± 2.36 years. From 1598 MII oocytes, 1,260 2  P N zygotes were obtained, yielding a fertilization rate of 78.85% (1,260/1,598). The cleavage rate was 98.49% (1,241/1,260), with 61.27% (772/1,260) of zygotes forming usable blastocysts and 45.79% (577/1,260) being high-quality. Of the 772 usable blastocysts underwent TE biopsy, 518 were subjected to NGS analysis, while the WGA products from the remaining 254 biopsied embryos were stored without immediate testing due to cost consideration. Conclusive NGS results were obtained for 516 blastocysts, revealing the following ploidy outcomes: 246 (47.67%) were euploid, 135 (26.16%) were aneuploid, and 135 (26.16%) exhibited mosaicism or segmental abnormalities (Table 1).

**TABLE 1:** Patient demographics and embryo development outcomes.

Characteristic	Value
Cycles (n)	111
Female age (years)	32.47 ± 3.83
BMI (kg/m^2^)	21.97 ± 2.65
Duration of infertility (years)	2.05 ± 2.36
Basal hormone levels	
FSH (IU/L)	6.66 ± 1.36
E2 (pmol/L)	154.49 ± 58.95
LH (IU/L)	5.93 ± 2.59
AMH (ng/ml)	3.98 ± 2.35
Ovarian stimulation	
Initial Gn dose (IU)	203.94 ± 34.17
Total Gn dose (IU)	2303.04 ± 631.19
Embryology outcomes	
Oocyte retrieved (n)	17.17 ± 6.04
Mature oocytes (n)	14.40 ± 5.05
Fertilization rate (%)	78.85 (1260/1598)
Cleavage rate (%)	98.49 (1241/1260)
D3 high-quality embryo rate (%)	50.08 (631/1260)
Usable blastocyst rate (%)	61.27 (772/1260)
High-quality blastocyst rate (%)	45.79 (577/1260)
Blastocysts biopsied (n)	772
Blastocysts subjected to NGS analysis (n)	518
Blastocysts with conclusive NGS results (n)	516
Ploidy analysis of outcome	
Euploidy rate (%)	47.67 (246/516)
Aneuploidy rate (%)	26.16 (135/516)
Mosaic or Segmental abnormality rate (%)	26.16 (135/516)

High-quality embryos, ≧ 6 cells with ≤ grade II fragmentation on day 3; usable blastocyst exhibited expansion ≥ 3 with ICM/TE grades of at least BC/CB; high-quality blastocysts demonstrated expansion ≥ 3 with ICM/TE grades of AA, AB/BA, or BB. Blastocysts graded ≥ 3 BC/CB were selected for TE biopsy.

### Impact of abnormal cleavage patterns on blastocyst formation and chromosomal ploidy

Among 1,241 cleaved embryos, 485 (39.08%) exhibited at least one abnormal cleavage pattern. The presence of DC, UB or BF was associated with significantly lower rates of both usable and high-quality blastocyst formation compared to embryos with normal cleavage (all *P < 0.001;* Table 2). Of the 516 blastocysts genetically diagnosed blastocysts, 139 (26.94%) were derived from abnormally cleaving embryos. Although embryos with DC or UB showed a trend toward lower euploidy rates, no abnormal cleavage pattern demonstrated a statistically significant effect on embryonic ploidy (all *P > 0.05;* Table 3).

**TABLE 2:** Impact of abnormal cleavage patterns on embryonic developmental potential.

Patterns	N	Usable blastocysts		High-quality blastocysts	
		% (n/N)	*P* value	% (n/N)	*P* value
Normal	756	73.68 (557/756)	-	58.47 (442/756)	-
DC	74	29.73 (22/74)	<0.001	13.51 (10/74)	<0.001
RC	5	40 (2/5)	0.120	40 (2/5)	0.654
UB	113	60.18 (68/113)	0.003	39.82 (45/113)	<0.001
Mn	96	67.71 (65/96)	0.215	53.13 (51/96)	0.318
BF	82	23.17 (19/82)	<0.001	4.88 (4/82)	<0.001

Data are presented as n (%). *P* values were derived from Chi-square test, except for groups with n < 5, where Fisher’s exact test was used. All tests are two-sided and compared to the Normal (reference) group. The analysis did not include 115 embryos due to the presence of mixed abnormal cleavage patterns (two or more types).

Abbreviations: DC, direct cleavage; RC, reverse cleavage; UB, uneven blastomere; Mn, multinucleation; BF, blastomere fragmentation.

**TABLE 3:** Impact of abnormal cleavage patterns on embryonic euploidy.

Patterns	Blastocysts biopsied (n)	Euploid (%)	*P* value
Normal	377	49.07 (185/377)	/
DC	12	33.33 (4/12)	0.262
RC	2	100.00 (2/2)	0.243
UB	43	37.21 (16/43)	0.14
Mn	45	46.66 (21/45)	0.760
BF	13	61.54 (8/13)	0.377

Data are presented as n (%). *P* values were derived from Chi-square test, except for groups with n < 5, where Fisher’s exact test was used. All tests are two-sided and compared to the Normal (reference) group. The analysis did not include 24 embryos due to the presence of mixed abnormal cleavage patterns (two or more types).

Abbreviations: DC, direct cleavage; RC, reverse cleavage; UB, uneven blastomere; Mn, multinucleation; BF, blastomere fragmentation.

### Kinetic parameters predict blastocyst formation but not ploidy

Embryos with a blastocyst grade of “CC” (n = 60) or developmental arrest (4 cells on day 3; n = 107) were excluded to ensure data quality. Analysis of the remaining embryos revealed that those forming usable blastocysts (n = 764) (8 blastocysts were excluded from kinetic analysis due to failed time-lapse image capture) exhibited significantly faster development across most kinetic parameters than those that did not (non-blastocysts; n = 302), except for t3 and t5 (all *P* < 0.05; Table 4). The optimal developmental range for each parameter was defined as the two consecutive quartiles associated with the highest usable blastocyst formation rate (Supplementary Table S1). Embryos developing within these optimal windows demonstrated significantly higher usable blastocyst formation rates (all *p < 0.001;* Supplementary Table S2).

**TABLE 4:** Comparison of parameters (hours) in usable blastocysts and non-blastocysts.

Parameter	Usable blastocysts	Non-blastocysts	*P* value
tPNf	24.10 (22.40, 25.80)	25.00 (22.70, 28.30)	<0.001
t2	26.35 (24.43, 28.50)	27.80 (25.18, 31.10)	<0.001
t3	37.70 (35.00, 40.70)	37.30 (32.68, 42.00)	0.267
t4	38.80 (36.13, 41.80)	40.60 (36.20, 45.53)	<0.001
t5	52.40 (48.10, 57.00)	51.25 (43.15, 58.60)	0.107
t8	58.70 (53.40, 64.10)	61.29 (57.80, 69.25)	<0.001
cc1	2.50 (2.20, 2.70)	2.50 (2.20, 2.90)	0.001
cc2	11.70 (10.80, 12.70)	11.60 (1.00, 12.90)	0.031
s2	0.50 (0.20, 1.18)	1.10 (0.28, 6.55)	<0.001
s3	4.20 (2.30, 10.30)	12.55 (4.50, 20.49)	<0.001

Data are presented as median (interquartile range). *P* values were calculated using the Mann-Whitney U test. The non-blastocyst subgroup exhibited skewed distributions (cc2: left-skewed; s2: right-skewed); all extreme values were verified against the raw data.

A multivariable logistic regression model incorporating these optimal-range kinetic parameters and abnormal cleavage patterns identified DC (OR = 0.209, *P* < 0.001) as negative predictors, and t8 (OR = 1.959, *P* = 0.006), s2 (OR = 1.603, *P* = 0.020), and s3 (OR = 1.967, *P* = 0.002) as positive predictors of usable blastocyst formation, whereas BF, UB, tPNf, t2, t4, cc1 and cc2 did not reach statistical significance in this model (Table 5). In the training set (70% of the data), the model demonstrated good predictive accuracy, with an AUC of 0.826 (95% CI: 0.792–0.860), with a sensitivity of 79.7% and a specificity of 75.1%.

**TABLE 5:** Generalized linear mixed model analysis for predicting usable blastocyst formation.

Parameter	OR (95%CI)	*P* value
tPNf	1.494 (0.971-2.297)	0.068
t2	1.129 (0.731-1.745)	0.584
t4	1.128 (0.710-1.791)	0.610
t8	1.959 (1.213-3.163)	0.006
cc1	1.317 (0.879-1.971)	0.182
cc2	1.274 (0.828-1.961)	0.270
s2	1.603 (1.079-2.381)	0.020
s3	1.967 (1.274-3.037)	0.002
DC	0.209 (0.118-0.368)	<0.001
UB	0.650 (0.404-1.047)	0.076
BF	0.432 (0.180-1.036)	0.060

OR, Odds ratio; CI, confidence interval.

When applied to the independent validation set (30% of the data), the model yielded an AUC of 0.724 (95% CI: 0.663–0.786), with a sensitivity of 71.6% and a specificity of 67.4% (Supplementary Figure S1). For comparison, the KID score generated by the TLI system was evaluated in the same validation set and achieved an AUC of 0.738 (95% CI: 0.677–0.799), with a sensitivity of 78.0% and a specificity of 60.7% (Supplementary Figure S2). The DeLong test comparing the two AUCs in the validation set showed no statistically significant difference (*P* = 0.682), indicating that our model and the KID score have comparable predictive performance for usable blastocyst formation (Table 6).

**TABLE 6:** Predictive performance of the model and KID score for usable blastocyst formation.

Dataset	Model	AUC (95%CI)	Sensitivity (%)	Specificity (%)	*P* value
Training	Our model	0.826 (95% CI: 0.792–0.860)	79.7	75.1	-
Validation	Our model	0.724 (95% CI: 0.663–0.786)	71.6	67.4	0.682
Validation	KID score	0.738 (95% CI: 0.677–0.799)	78.0	60.7	Reference

*P* value from DeLong test comparing our model vs. KID score in the validation set. AUC, area under the curve; CI, confidence interval.

In contrast, comparison of a genetically pure euploid cohort (n = 246) and a pure aneuploid cohort (n = 135) revealed no significant differences in any of the 10 morphokinetic parameters (all *P > 0.05*; Supplementary Table S3). Univariate logistic regression analysis confirmed that no kinetic parameter was associated with embryonic euploidy (all ORs≈1.0, all *P > 0.05*; Table 7).

**TABLE 7:** Logistic regression analysis of morphokinetic parameters for predicting embryo euploidy.

Parameter	OR (95%CI)	*P* value
tPNf	0.991 (0.912-1.077)	0.832
t2	0.993 (0.924-1.068)	0.857
t3	1.019 (0.969-1.071)	0.468
t4	1.012 (0.962-1.064)	0.652
t5	0.997 (0.965-1.030)	0.845
t8	0.987 (0.962-1.013)	0.309
cc1	0.973 (0.928-1.021)	0.262
cc2	1.040 (0.973-1.111)	0.249
s2	0.984 (0.910-1.063)	0.674
s3	0.984 (0.953-1.016)	0.321

OR, Odds ratio; CI, confidence interval.

### Metabolomic profiling and exploratory candidates for embryonic euploidy

Metabolomic profiling was performed on 27 pooled samples (euploid group:17 or aneuploid group:10). A total of 785 metabolites were identified. Data reliability was confirmed by quality control analysis, with >70% of peaks exhibiting a RSD ≤0.3 (Supplementary Figure S3). Multivariate analyses revealed consistent metabolic separation between euploid and aneuploid groups, as evidenced by PCA (Supplementary Figure S4), PLS-DA (Supplementary Figure S5) and OPLS-DA (Figure 2). Permutation testing confirmed the robustness, predictive validity, and absence of overfitting in these models (Supplementary Figures S6, S7).

Based on the OPLS-DA model (VIP> 1.0, *P < 0.05*), 86 metabolites were identified, with 46 upregulated, and 40 downregulated in the euploid group (Figure 3). KEGG pathway enrichment analysis based on these 86 nominally significant features revealed that these metabolites were significantly enriched in pathways representing three key functional categories: cellular infrastructure and nutrition (protein digestion and absorption, branched-chain amino acid degradation), sphingolipid signaling and metabolism, and biomolecular synthesis fidelity (aminoacyl-tRNA biosynthesis) (Figure 4).

**FIGURE 3:**
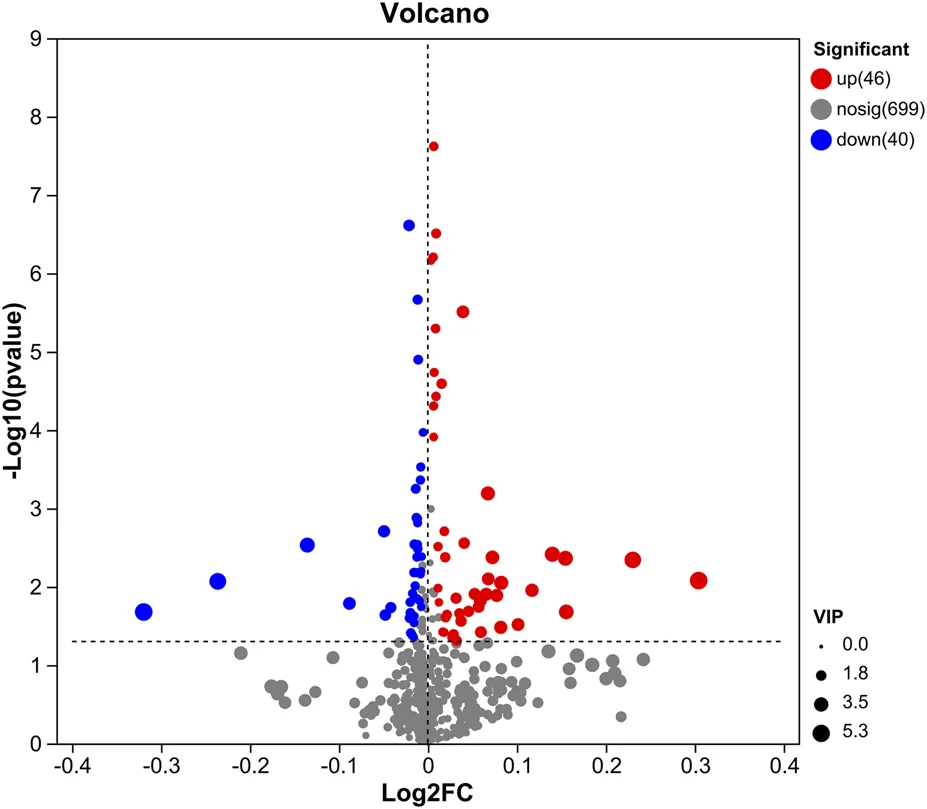
Volcano plot of differentially abundant metabolites between euploid and aneuploid embryos. Note: The horizontal axis represents the fold change (FC) in the expression of the metabolites between the two groups, i.e.,log 2 F C, and the vertical axis represents the statistical significance of the change in metabolite expression,i.e.,-log10 (p_value). Red and blue points indicate significantly upregulated and downregulated metabolites (VIP>1, *P* < 0.05). Gray points represent non-significant metabolites.

**FIGURE 4:**
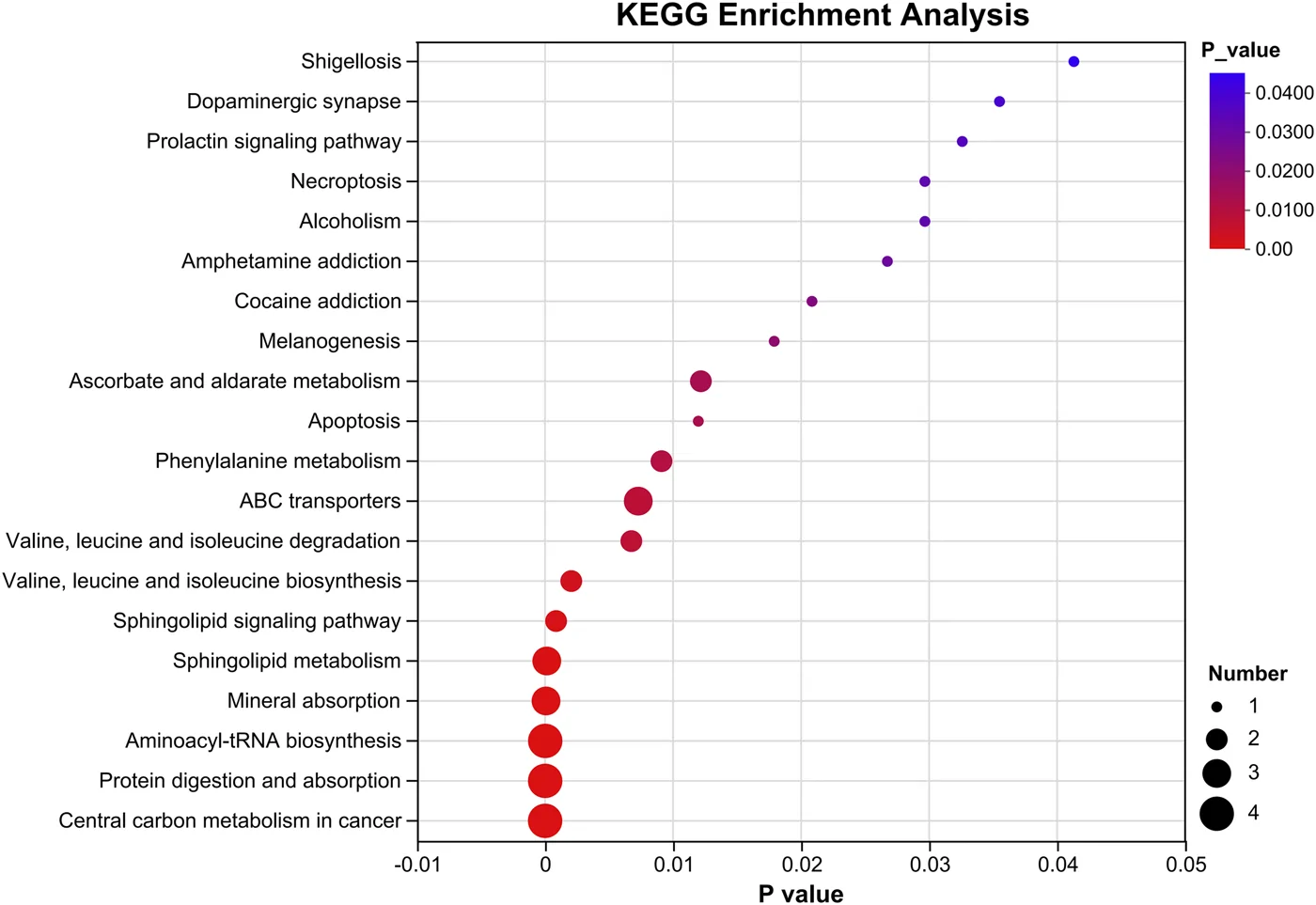
KEGG pathway enrichment analysis of differential metabolites associated with embryonic euploidy. Note: Top significantly enriched pathways are shown (*P* < 0.01), with point size representing the number of metabolites per pathway and color indicating enrichment significance.

To prioritize biomarkers relevant to endogenous metabolism, we retained 11 metabolites from the 86 VIP-ranked features (Supplementary Table S4). Consistent with the multiple-testing control applied, none reached significance after Benjamini–Hochberg FDR correction (FDR-adjusted *P > 0.05*). Nevertheless, given that kinetic parameters showed no association with ploidy, we assessed the exploratory utility of these metabolites. ROC analysis revealed that six metabolites—sphingosine, phytosphingosine, N,N-dimethylsphingosine, histidylproline, L-valine, and indoleacetaldehyde—exhibited AUC >0.7 (Figure 5), albeit based on uncorrected P-values. Of note, the fold changes of these candidate metabolites ranged from 0.988 to 1.042 (corresponding to −1.2% to +4.2%), indicating relatively small effect sizes; therefore, fold-change was not employed as a filtering criterion in this selection process. We emphasize these as hypothesis-generating findings that require prospective validation in non-pooled, larger sample sets.

**FIGURE 5:**
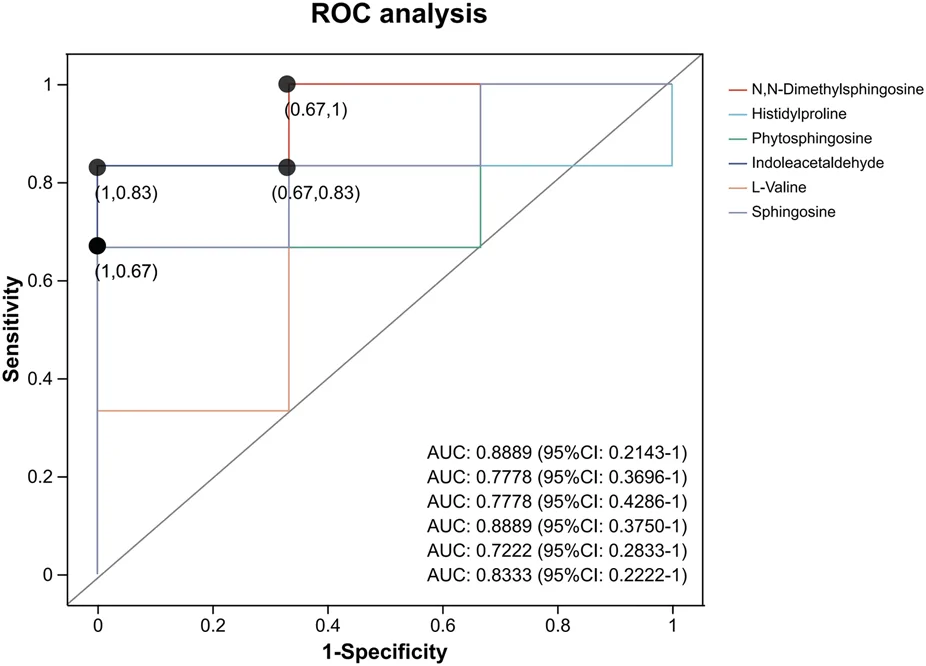
Receive operating characteristic (ROC) curve of the six metabolite predictors for discriminating embryonic euploidy. Note: ROC analysis was based on 17 euploid pools vs. 10 aneuploid pools.

## Discussion

The primary goal of ART is the birth of a healthy singleton, and eSET is the most effective strategy to achieve this objective (Mejia et al., 2020). Currently, eSET can be performed either at the blastocyst stage or at the cleavage stage. While blastocyst transfer offers higher implantation rates, extended culture to D5 carries inherent risk, including culture failure and concerns over epigenetic and telomere-related effects (White et al., 2015; Sirard, 2017). By contrast, D3 transfer avoids these risks and is particularly valuable for older patients, those with low oocyte yield, or those with a limited number of good-quality embryos. However, its main drawback is relatively lower implantation rate. Thus, if a single cleavage stage embryo could achieve implantation rates comparable to those of a blastocyst, D3 transfer would become a favorable alternative, highlighting the need for reliable early biomarkers of developmental competence. In this context, TLI has emerged as a valuable, non-invasive tool for assessing developmental potential through morphokinetic; however, its utility in predicting embryonic ploidy remains a subject of considerable debate. Previous studies have reported conflicting conclusions. While some indicate that kinetic parameters correlate with embryo ploidy and possess predictive value (Basile et al., 2015; Chawla et al., 2015; Del Carmen Nogales et al., 2017; Campbell et al., 2013), other refuting (Zhang J. et al., 2017; Rienzi et al., 2015; Kramer et al., 2014; Reignier et al., 2018) such an association, highlighting the absence of a clear consensus and underscoring the need for further investigation.

Our study revealed that abnormal cleavage patterns and early kinetic parameters significantly predicted blastocyst formation, but showed no association with embryonic ploidy. A GLMM integrating these parameters demonstrated good predictive performance for blastocyst formation (training AUC = 0.826; validation AUC = 0.724), comparable to the KID score (validation AUC = 0.738; DeLong P = 0.682). This suggests that the predictive power of kinetic parameters is confined to blastocyst formation potential in cleavage-stage embryos and does not encompass the further prediction of euploidy. Our conclusions align directly with those of Desai et al., who demonstrated that abnormal cleavage patterns and suboptimal kinetic parameters impaired blastocyst formation without affecting euploidy rates (Desai et al., 2018). Similarly, Pennetta et al. reported a strong association between the s2 parameter and blastocyst quality but found no link between most kinetic parameters and ploidy, with the notable exception of the pronuclei appearance time (tPNa) (Pennetta et al., 2021). It is worth noting that tPNa was not analyzed in our study because embryos were loaded into the time-lapse system on D1 only after confirmation of 2  P N.

In contrast, few studies have reported divergent findings. Bamford et al. identified later-stage timings (t8, t9, tB, tEB) as the most significant variables for predicting euploidy (Bamford et al., 2022). Furthermore, Huang et al. employed time-lapse imaging to capture kinetic parameters from 1,803 biopsied blastocysts and developed an artificial intelligence-based euploid prediction algorithm that achieved an AUC of 0.80 (Huang et al., 2021). These divergent findings may be attributed to differences in the developmental stages analyzed, patient populations, or methodological approaches. The persistent inconsistency across studies suggests that the predictive value of kinetics for ploidy is highly context-dependent and is not yet a universally reliable, stand-alone tool.

In parallel, metabolomic profiling of SCM has emerged as a promising alternative. Building on our previous work (Chen et al., 2025a; Chen et al., 2025b), we performed LC-MS-based metabolomic analysis of D3 SCM from euploid and aneuploid embryos. PCA, PLS-DA, and OPLS-DA revealed significant metabolic disparities between the two groups, with permutation tests confirming model robustness. Based on the OPLS-DA model, we prioritized 86 features (VIP>1.0, *P* < 0.05). However, after Benjamini–Hochberg FDR correction, none retained statistical significance (all adjusted *P* > 0.05). These findings are therefore exploratory and hypothesis-generating, with all candidate metabolites annotated with FDR-adjusted *P*-value in Supplementary Table S4. KEGG enrichment analysis of the 86 nominally significant features revealed involvement in amino acid and sphingolipid metabolism pathways. These pathways have been associated with embryonic development in other biological systems (Zhang S. et al., 2017; Martin et al., 2001; Gangloff et al., 2004; Guertin et al., 2006; Iyer et al., 2024), but their relevance to our findings remains speculative.

Given the exploratory nature of these results and the absence of direct evidence in human embryos, mechanistic interpretations should be considered highly speculative and hypothesis-generating. The observed trends in sphingolipid metabolites (sphingosine, phytosphingosine, N,N-dimethylsphingosine), L-valine, indoleacetaldehyde, and histidylproline may suggest potential links to bioactive lipid signaling, BCAA/mTOR, AhR, and antioxidant pathways (Futerman, 2024; Ruiz et al., 2022; Guo et al., 2014; Ha et al., 2024; Vasconcelos et al., 2017; Shan et al., 2025; Wang et al., 2023; Eckert et al., 2012; Zablon et al., 2021; Minelli et al., 2009), but none have been validated in embryonic models and require direct experimental confirmation.

To explore clinical utility, we screened 11 endogenous metabolites and evaluated their predictive performance for ploidy. Six metabolites showed tentative discriminatory trends (AUC>0.7, based on 17 euploid and 10 aneuploid pooled samples) based on uncorrected *P*-values. Given that these AUC values were derived from pooled samples, they may overestimate true embryo-level performance. We therefore describe these as exploratory candidates requiring independent validation in larger, non-pooled cohorts.

Our findings align with the emerging recognition of metabolomics’ potential in embryo selection. However, robust studies in this area remain limited. For instance, Liang et al. (Liang et al., 2019) employed Raman spectroscopy to analyze the D5 SCM and revealed that chromosomal abnormalities alter its metabolic footprint, suggesting that profiling these shifts can predict embryonic ploidy. In a separate investigation, Eldarov et al. (Eldarov et al., 2022) conducted LC-MS analysis on D5 SCM and demonstrated clear sample clustering based on karyotype, with most significantly enriched metabolic pathways being associated with amino acid, fatty acid, and vitamin metabolism. Collectively, these findings suggest that metabolomic profiling may hold greater promise than morphokinetic parameters for the non-invasive prediction of embryonic euploidy. Notably, the metabolic evidence in our study was derived from D3 SCM, indicating that chromosomal status may be assessed at an earlier developmental stage, though this observation requires prospective confirmation.

While this study delineates the distinct roles of morphokinetics and metabolomics in embryo assessment, several limitations merit consideration. First, the generalizability of our findings is limited by the strict inclusion criteria (age≤38 years, ≥8 oocytes retrieved, ≥5 2  P N zygotes) and the single-center PGT-M/PGT-A population. The conclusions primarily apply to young women (≤38 years) with normal ovarian function. For patients of advanced age (>38 years) or those with poor ovarian response (e.g., <8 oocytes retrieved), their embryonic characteristics and response to interventions remain unclear. Therefore, direct extrapolation of our conclusions to advanced-age, poor-responders, or non-PGT populations should be undertaken with caution. Future multicenter prospective studies are needed to validate the external validity of our findings across different subgroups. Second, all morphokinetic annotations were performed by a single experienced embryologist, albeit prospectively and blinded to outcomes. Although predefined criteria were applied and annotations were periodically reviewed by a second senior embryologist, formal inter- and intra-observer agreement were not calculated. This may introduce subjectivity and limit reproducibility. Future studies with independent double annotation and formal reliability assessments would strengthen the generalizability of our findings. Third, the metabolomic analysis was restricted to euploid and aneuploid embryos, excluding mosaic and segmentally abnormal embryos, thus leaving their metabolism uncharacterized. Fourth, to ensure the analytical reliability of non-targeted metabolomics, the storage duration of all samples was strictly controlled within 3 months, which limited the total number of SCM samples collected in this study. Because a single embryo yields only 25 µL of SCM, which is below the 100 µL required by our LC-MS platform, we pooled four samples to meet detection sensitivity. However, this pooling strategy unavoidably compromises embryo-level resolution, masks metabolic heterogeneity, and may overestimate the performance of biomarker candidates in ROC analyses, as the AUC estimates were derived from 17 euploid and 10 aneuploid pooled samples rather than from individual embryos. Consequently, our metabolomic findings are hypothesis-generating and cannot support embryo-level clinical decisions. Although we strictly controlled for inclusion/exclusion criteria and culture conditions to minimize confounding, we did not formally adjust for potential confounders such as BMI or semen quality, which may have influenced the metabolomic profiles. Therefore, validation on a more sensitive platform with non-pooled designs is required. Fifth, due to the limited sample size of embryos and SCM, our current study is confined to exploring the independent predictive value of kinetic parameters and metabolomics for embryonic developmental potential, and has not yet integrated the two to construct a comprehensive predictive model. Sixth, formal calibration assessment for the mixed model was not performed in this exploratory analysis, which we acknowledge as a limitation.

Moreover, because metabolomic profiling was performed on pooled SCM samples, direct integration with embryo-level morphokinetic data was not methodologically feasible. This will be a priority in our future studies, where we plan to expand the sample size and integrate embryo kinetics with non-pooled metabolomics to construct a comprehensive non-invasive predictive model for embryo developmental potential. This represents a critical and promising direction for subsequent research in this field.

## Conclusion

In summary, our findings suggest that early kinetic parameters and D3 SCM metabolomics provide distinct and potentially complementary information for embryo selection. Morphokinetics may help predict blastocyst formation (validation AUC = 0.724), while metabolomic profiling offers exploratory metabolic clues associated with ploidy status, though these findings require prospective validation in non-pooled, independent cohorts. Together, these results lay a preliminary foundation for a future integrated non-invasive assessment strategy on Day 3.

## Data Availability

The raw data supporting the conclusions of this article will be made available by the authors, without undue reservation.
