# Supplementary material for: Non-invasive assessment of embryo development and ploidy: morphokinetics predict blastulation, and exploratory metabolomics identifies candidate metabolic signatures for euploidy
Source: Front Cell Dev Biol. 2026 Sep 18;14:1914948. doi: 10.3389/fcell.2026.1914948 (PMC13631084; doi:10.3389/fcell.2026.1914948)
Supplement: Supplementary file 1 [file DataSheet1.pdf]

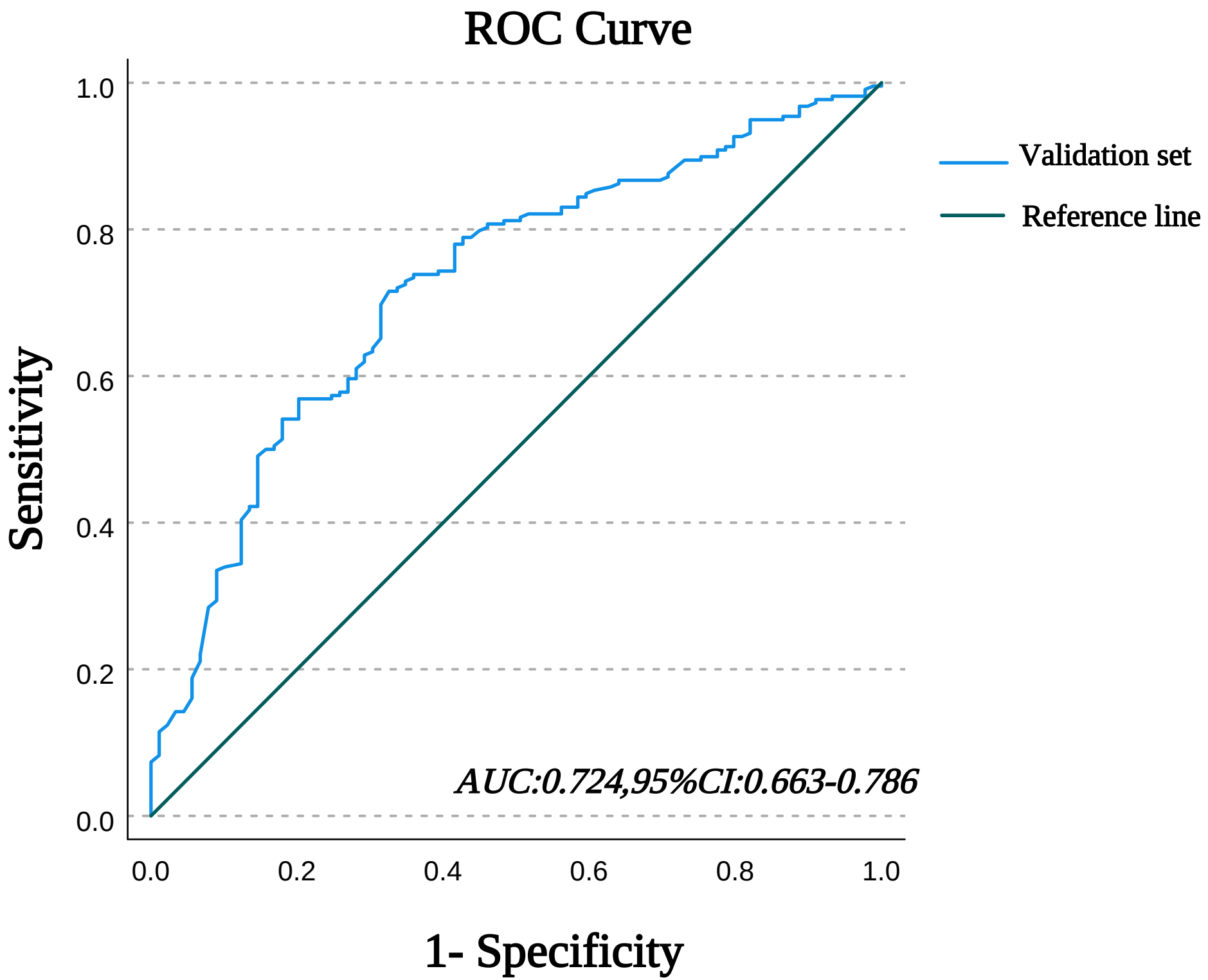

Supplemental Figure 1 ROC curve analysis of kinetic parameters for predicting blastocyst formation

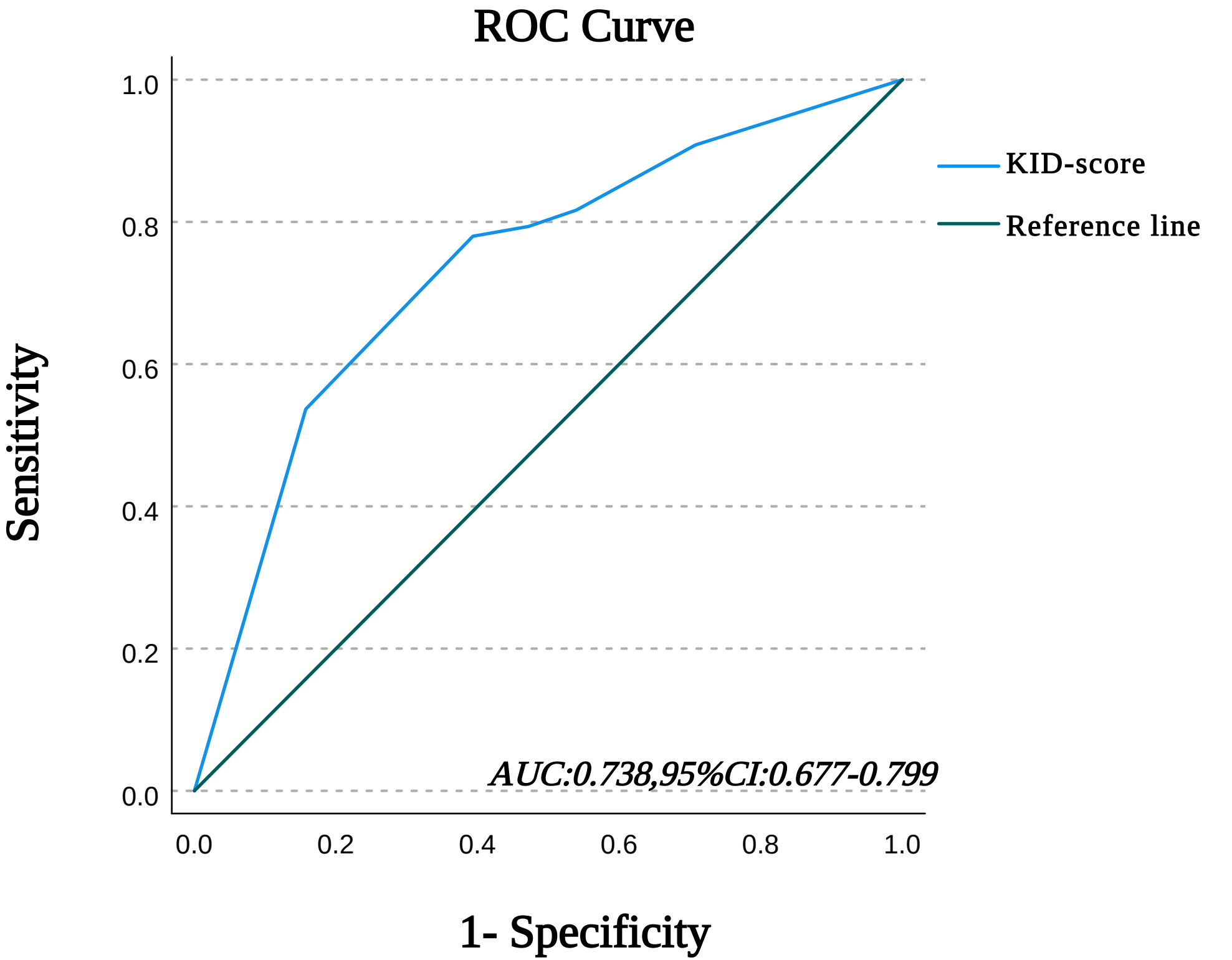

Supplemental Figure 2 ROC curve analysis of KID score for predicting blastocyst formation .

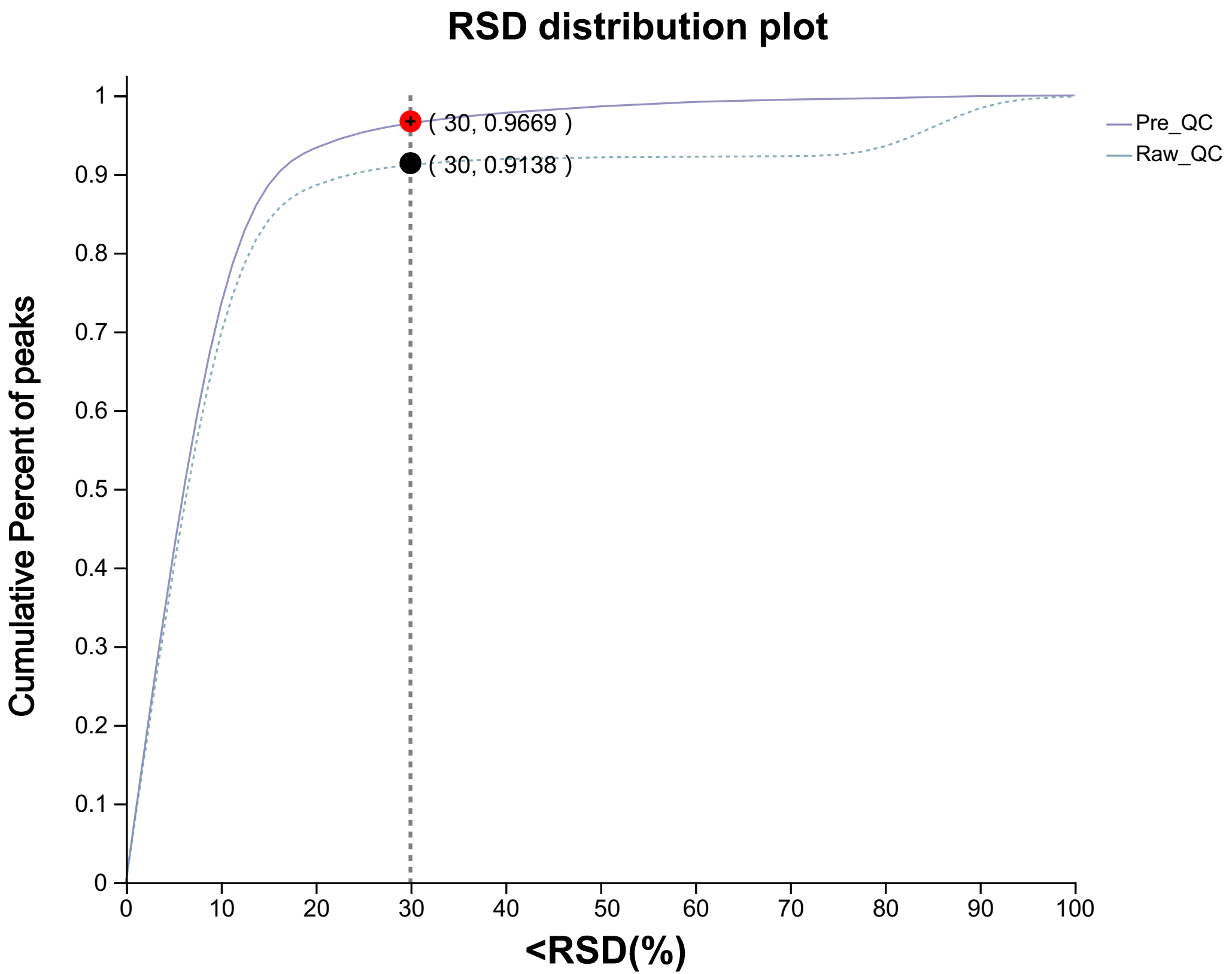

Supplemental Figure 3 QC assessment of metabolomics data using RSD distribution .
Note:The horizontal axis represents the RSD (%) value, which is the standard deviation divided by the mean, and the vertical axis represents the cumulative proportion of the ion peak.Dashed and solid lines indicate pre- and post- processing data, respectively.

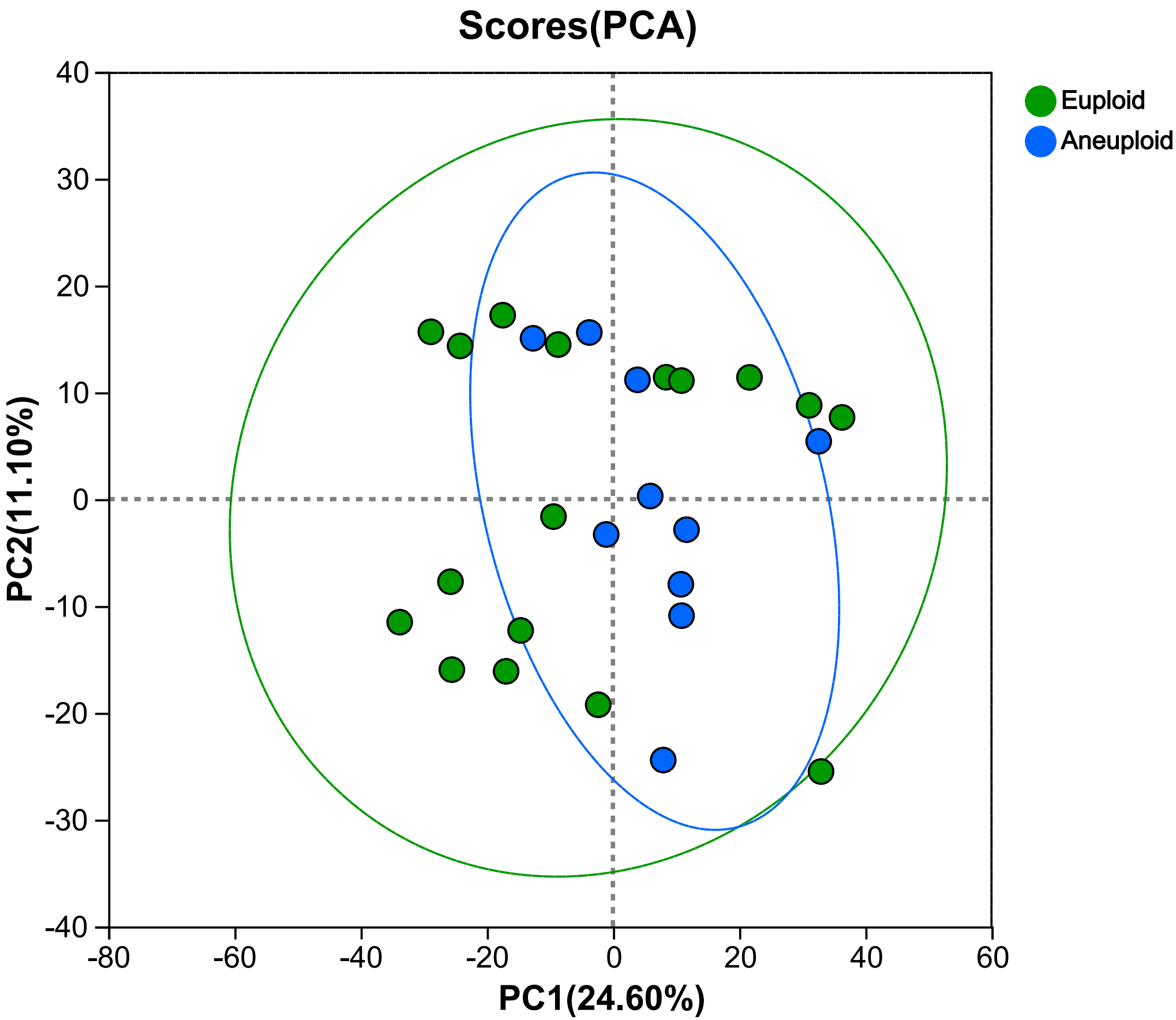

Supplemental Figure 4 PCA score plot of embryo D3SCM metabolomes

Note:PC1 (24.60%) and PC2 (11.10%) represent the contribution values of the first and second principal components, respectively.Points represent individual SCM grouped by embryonic euploid (green: Euploid; blue: Aneuploid).

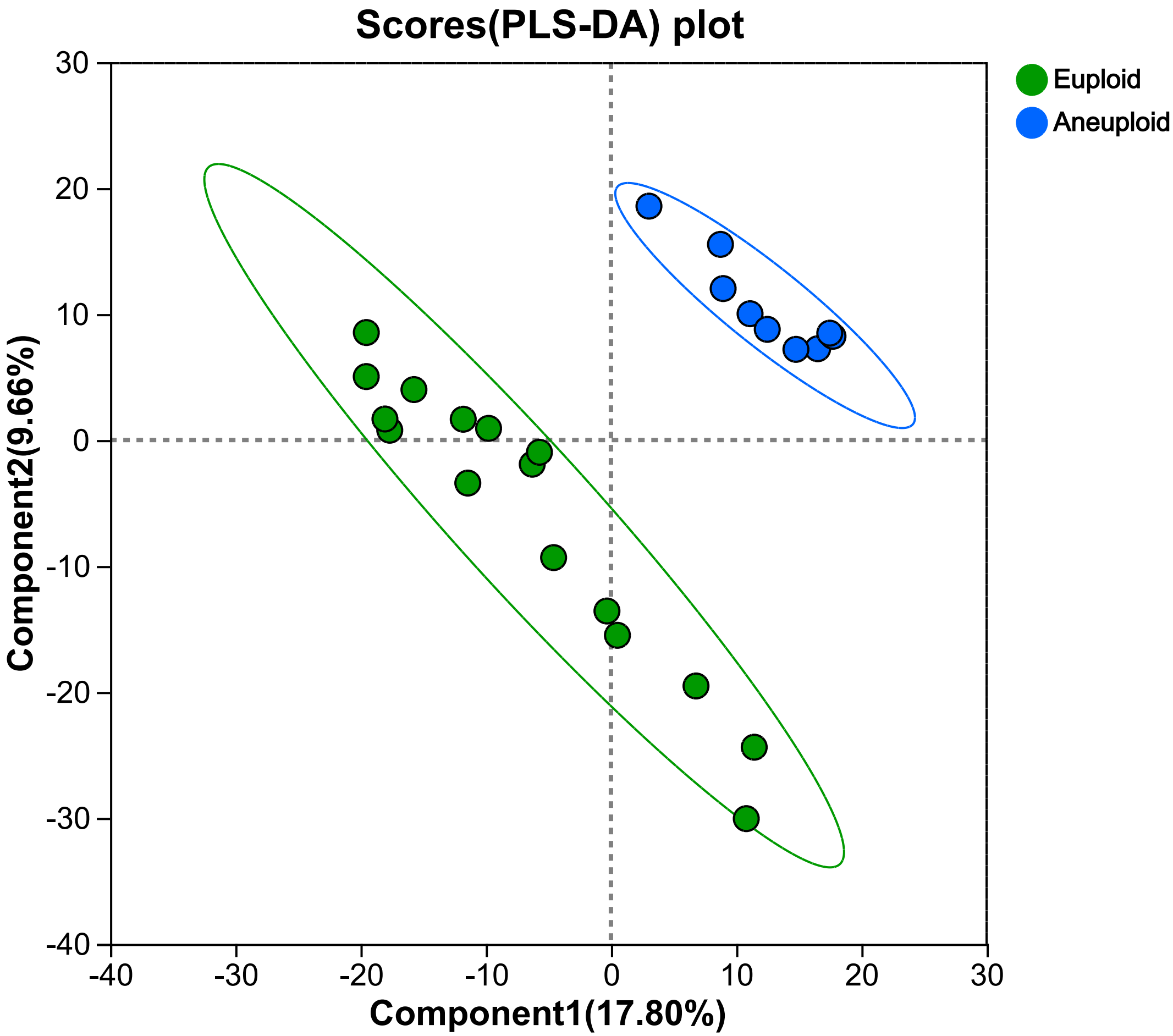

Supplemental Figure 5 PLS-DA score plot of embryo D3SCM metabolomes

Note:Component 1 (17.80%) and Component 2 (9.66%) represent the explained variance of the first and second principal components, respectively. Points represent individual SCM grouped by embryonic euploid(green: Euploid; blue: Aneuploid).

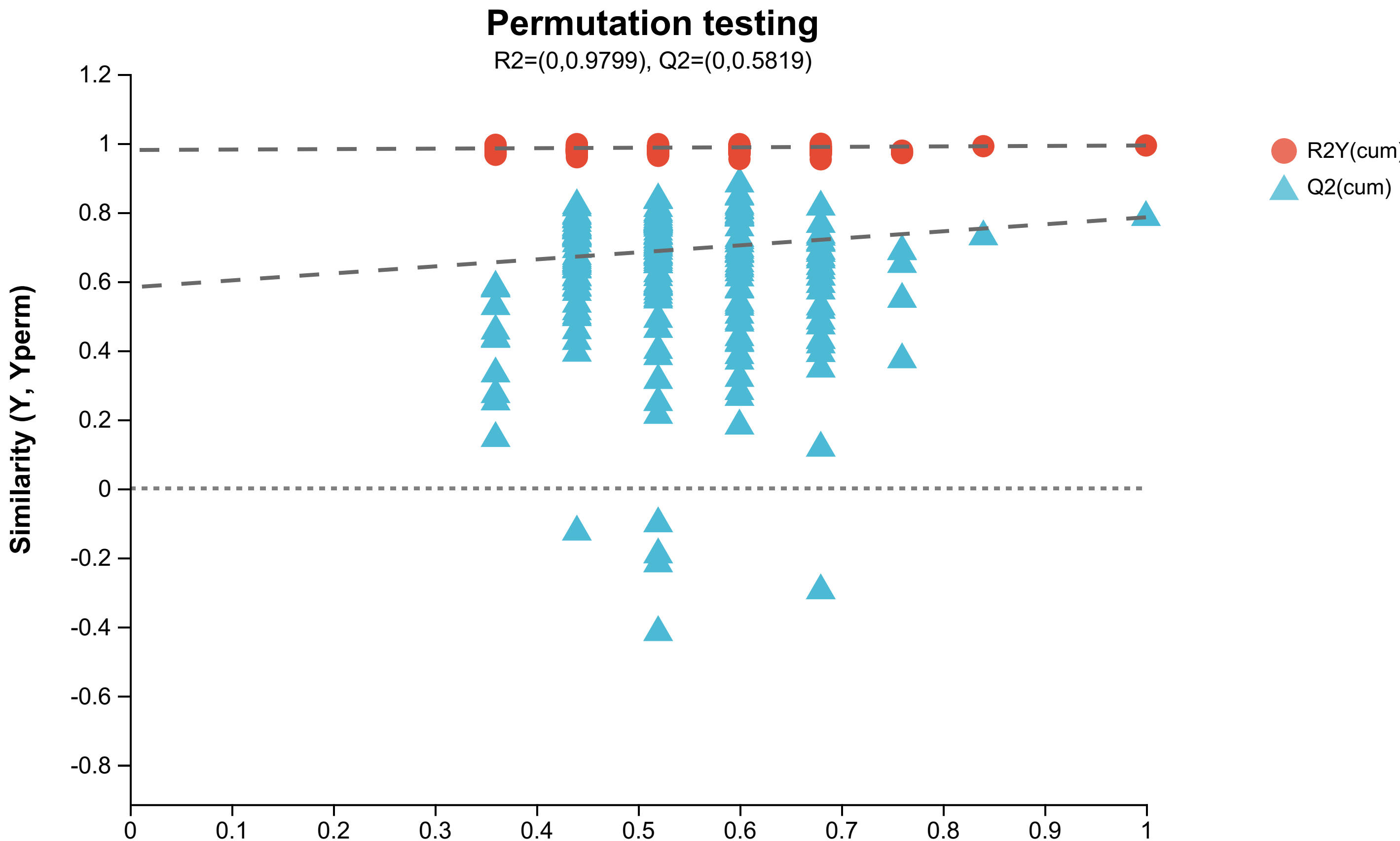

Supplemental Figure 6 Validation of the PLS-DA model by permutation testing

Note:The x-axis indicates the permutation retention degree of the permutation test; the y-axis shows the values of R^2(blue circles) and Q^2(red triangles) from the permutation test. The two dashed lines represent the regression lines for $R^2$and Q^2, respectively.

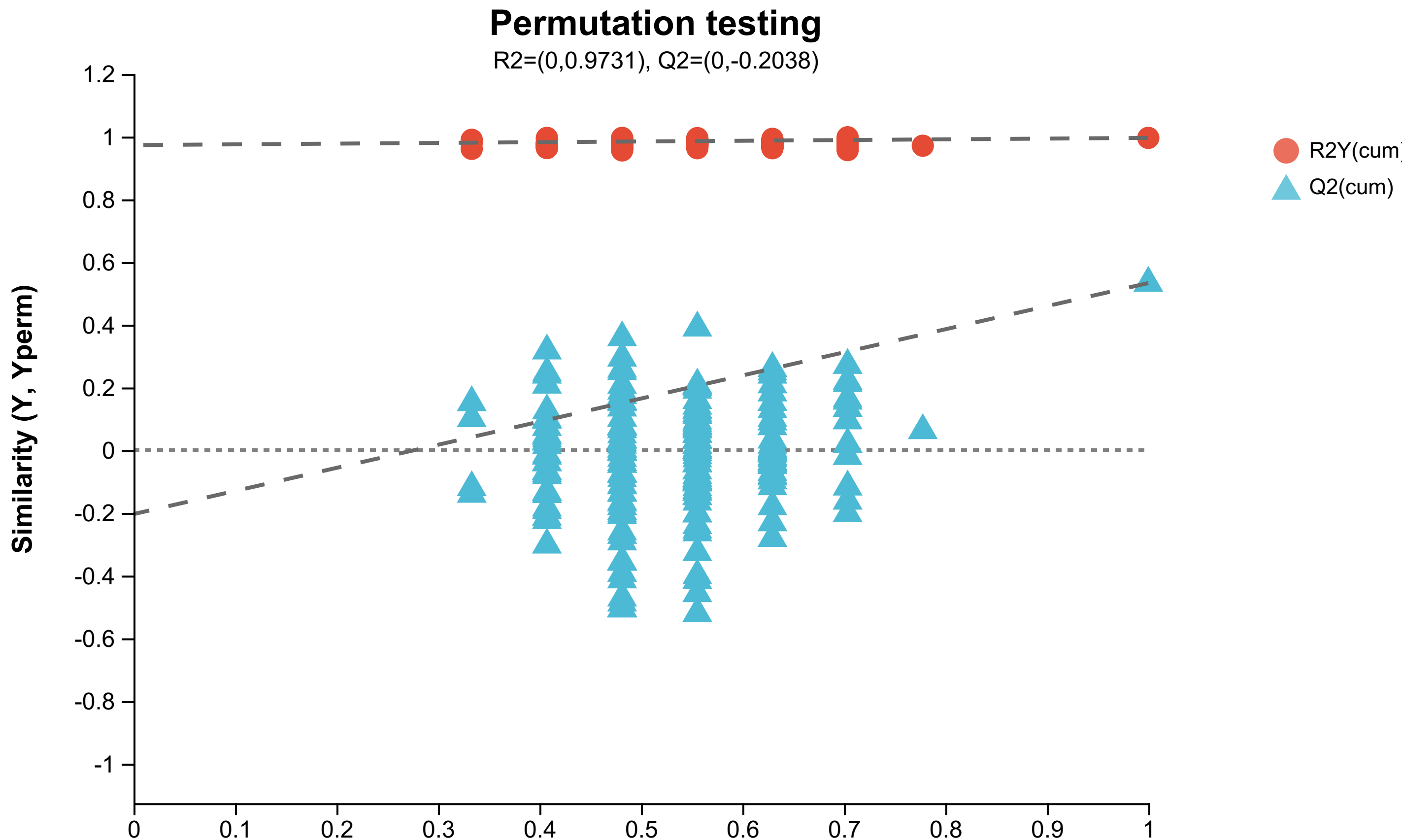

Supplemental Figure 7 Validation of the OPLS-DA model by permutation testing

Note:The x-axis indicates the permutation retention degree of the permutation test; the y-axis shows the values of R^2 (blue circles) and Q^2(red triangles) from the permutation test. The two dashed lines represent the regression lines for $R^2$and Q^2, respectively.
