# Supplementary material for: Non-invasive assessment of embryo development and ploidy: morphokinetics predict blastulation, and exploratory metabolomics identifies candidate metabolic signatures for euploidy
Source: Front Cell Dev Biol. 2026 Sep 18;14:1914948. doi: 10.3389/fcell.2026.1914948 (PMC13631084; doi:10.3389/fcell.2026.1914948)
Supplement: Supplementary file 2 [file Table1.doc]

| **Supplemental Table1 Comparison of usable blastocyst formation rates after stratifying morphokinetic parameters into quartiles (Q1, Q2, Q3, and Q4).** | | | | | | | | | |
| --- | --- | --- | --- | --- | --- | --- | --- | --- | --- |
| **Parameter** | **Q1** | | **Q2** | | **Q3** | | **Q4** | | ***P value*** |
|  | Limit(h) | Usable blastocyst(%) | Limit(h) | Usable blastocyst(%) | Limit(h) | Usable blastocyst(%) | Limit(h) | Usable blastocyst(%) |  |
| tPNf | <22.50 | 76.10(201/264) | **[22.50-24.30]** | 77.80(210/270) | **(24.30-26.40]** | 77.00(207/269) | >26.40 | 55.50(146/263) | *<0.001* |
| t2 | **<24.60** | 79.40(200/252) | **[24.60-26.80]** | 76.40(223/292) | (26.80-29.10] | 75.30(195/259) | >29.10 | 55.50(146/263) | *<0.001* |
| t4 | <36.20 | 72.90(191/262) | **[36.20-39.10]** | 81.10(223/275) | **(39.10-42.70]** | 75.90(202/266) | >42.70 | 56.30(148/263) | *<0.001* |
| t8 | **<54.30** | 85.40(223/261) | **[54.30-60.35]** | 80.90(220/272) | (60.35-64.80] | 54.50(146/268) | >64.80 | 66.60(175/265) | *<0.001* |
| cc1 | **<2.20** | 71.70(167/233) | **[2.20-2.50]** | 77.60(337/434) | (2.50-2.70] | 68.90(93/135) | >2.70 | 63.30(167/264) | *<0.001* |
| cc2 | <10.50 | 56.40(145/257) | **[10.50-11.70]** | 84.60(259/306) | **(11.70-12.70]** | 76.30(190/249) | >12.70 | 66.90(170/254) | *<0.001* |
| s2 | <0.20 | 67.40(89/132) | **[0.20-0.60]** | 81.10(344/424) | **(0.60-1.50]** | 79.80(202/253) | >1.50 | 50.20(129/257) | *<0.001* |
| s3 | **<2.50** | 82.50(212/257) | **[2.50-5.20]** | 85.90(232/270) | (5.20-14.79] | 68.10(186/273) | >14.79 | 50.40(134/266) | *<0.001* |
| Note: Numbers in bold indicate the two quartiles with the highest available blastocyst rate .*P* value were calculated by Chi-square test. | | | | | | | | | |

| **Supplemental Table 2 Comparison of usable blastocyst formation rates within vs. outside optimal range of morphokinetic parameters.** | | |
| --- | --- | --- |
| **Parameter** | **Usable blastocyst(%)** | ***P value*** |
| tPNf [22.50-26.40] | 77.37(417/539) | *<0.001* |
| Outside range | 65.84(347/527) |  |
| t2≤26.80 | 77.76(423/544) | *<0.001* |
| Outside range | 65.33(341/522) |  |
| t4 [36.20-42.70] | 78.56(425/541) | *<0.001* |
| Outside range | 64.57(339/525) |  |
| t8≤60.35 | 83.11(443/533) | *<0.001* |
| Outside range | 60.23(321/533) |  |
| cc1≤2.50 | 75.56(504/667) | *<0.001* |
| Outside range | 65.16(260/399) |  |
| cc2 [10.50-12.70] | 80.90(449/555) | *<0.001* |
| Outside range | 61.64(315/511) |  |
| s2 [0.2-1.50] | 80.65(546/677) | *<0.001* |
| Outside range | 56.04(218/389) |  |
| s3≤5.20 | 84.25(444/527) | *<0.001* |
| Outside range | 59.37(320/539) |  |
| Note: *P value were calculated by Chi-square test.* | | |

| Supplemental Table 3 Comparison of morphokinetic parameters (hours) between euploid and aneuploid embryos. | | | |
| --- | --- | --- | --- |
| **Parameter** | **Euploid(n=246)** | **Aneuploid(n=135)** | **P value** |
| tPNf | 23.80(22.10,25.70) | 23.99(22.30,25.40) | 0.627 |
| t2 | 26.10(24.20,28.30) | 26.30(24.20,28.00） | 0.845 |
| t3 | 37.10(34.88,40.63) | 37.42(34.90,39.30) | 0.691 |
| t4 | 38.10(35.80,41.40) | 38.40(35.70,40.30) | 0.656 |
| t5 | 52.10(47.98,56.40) | 52.10(48.70,55.90) | 0.925 |
| t8 | 57.15(52.38,62.80) | 58.44(53.20,63.50) | 0.249 |
| cc1 | 2.50(2.20,2.80) | 2.50(2.30,2.80) | 0.510 |
| cc2 | 11.55(10.78,12.70) | 11.40(10.70,12.30) | 0.191 |
| s2 | 0.50(0.20,1.03) | 0.40(0.20,0.90) | 0.526 |
| s3 | 3.50(2.20,6.39) | 4.50(2.60,7.80) | 0.064 |
| Note:Data are presented as median (interquartile range). P values were calculated using the Mann-Whitney U test. | | | |

| **Supplemental Table 4 Exploratory metabolites prioritized by OPLS-DA (VIP >1.0) with *P* < 0.05.** | | | | | |
| --- | --- | --- | --- | --- | --- |
| **Metabolite name** | **VIP** | **FC** | **Log₂FC** | ***P*** | **FDR-adjusted *P-value*** |
| Ascorbic Acid | 5.321 | 0.801 | -0.319 | 0.021 | 0.332 |
| Phytosphingosine | 2.800 | 1.042 | 0.059 | 0.015 | 0.290 |
| Sphingosine | 2.376 | 1.037 | 0.053 | 0.012 | 0.267 |
| N,N-Dimethylsphingosine | 2.298 | 1.042 | 0.060 | 0.038 | 0.411 |
| L-Proline | 1.624 | 0.986 | -0.020 | 0.025 | 0.352 |
| Sphinganine | 1.537 | 1.015 | 0.022 | 0.023 | 0.342 |
| Cysteinyl-Asparagine | 1.307 | 0.992 | -0.011 | 0.003 | 0.134 |
| Histidylproline | 1.302 | 0.988 | -0.017 | 0.042 | 0.423 |
| L-Isoleucine | 1.212 | 1.013 | 0.018 | 0.038 | 0.411 |
| L-Valine | 1.175 | 0.990 | -0.015 | 0.024 | 0.348 |
| Indoleacetaldehyde | 1.001 | 0.995 | -0.008 | 0.018 | 0.313 |
| Note: Metabolites were selected based on VIP > 1.0 and nominal P < 0.05 from the OPLS-DA model. FDR-adjusted *P-value* were calculated using the Benjamini-Hochberg procedure for 785 features; none of the listed metabolites reached statistical significance at FDR-adjusted *P* < 0.05. Log₂FC values represent the magnitude and direction of change (negative = lower abundance in the euploid group; positive = higher abundance in the euploid group). These metabolites are presented as exploratory candidates requiring independent validation. | | | | | |
